# Rate of Dental Extractions in Patients with Sickle Cell Disease

**DOI:** 10.3390/jcm11206174

**Published:** 2022-10-19

**Authors:** Adeel Ahmad, Diana Mihalca, Ben Stacey, Sayna Samaee, Dipal Mehta, Stephen Hibbs, Tanya Freeman, Basabi Chatterjee, Enamul Ali, Leo Cheng, Dimitris A. Tsitsikas

**Affiliations:** 1 Haemoglobinopathy Service, Department of Haematology, Homerton University Hospital NHS Foundation Trust, London E9 6SR, UK; 2 Department of Oral & Maxillofacial, Head & Neck Surgery, Homerton University Hospital NHS Foundation Trust, London E9 6SR, UK

**Keywords:** sickle cell disease, dental health, dental extraction

## Abstract

Background: Sickle cell disease is an inherited disorder associated with chronic haemolysis and anaemia, recurrent episodes of pain and potentially multisystem end-organ damage. A lot less is known about the dental health of these patients. Aims: To explore the incidence of severe dental disease leading to dental extraction in our sickle cell population. Patient/methods: We undertook an audit looking at the rate of dental extractions, as a composite marker of severe dental disease, among sickle cell patients over a 3-month period. The patients were unselected and approached during routine assessments. We analysed both clinical and laboratory data to look for possible associations between dental disease and sickle cell characteristics. Results: 177 patients were interviewed between February 2022 and April 2022. Overall, 71% of the patients had at least one dental extraction with a median number of teeth extracted of three and a median age at first extraction of 26. More than half of the patients stated that they do not have regular dental check-ups. There were no significant associations with the severity of sickle cell phenotype, baseline Hb or markers of haemolysis. Conclusion: A large number of patients with sickle cell disease require dental extractions at a relatively young age. The lack of any correlation with disease severity suggests that poor engagement with dental services and the underestimation of the importance of dental health are the main factors behind the increased prevalence of severe dental disease. Actively enquiring about dental problems should be part of any routine consultation with these patients, both in primary and specialist care.

## 1. Introduction

Sickle cell disease (SCD) is the most common inherited disorder in the UK, resulting from the substitution of glutamate by valine in position 6 of the β globin gene and giving rise to abnormal haemoglobin or “sickle haemoglobin” (HbS). HbS molecules within the red cell tend to polymerise under certain conditions such as inflammation, hypoxia, acidosis or dehydration, giving rise to the characteristic “sickle” shape of the erythrocytes [1]. These deformed and rigid erythrocytes lead to the two cardinal manifestations of SCD: a) vaso-occlusion due to impaired rheology and infarction of the microvasculature and b) haemolysis and chronic anaemia due to red cell deformability and the increased destruction of both intra- and extra-vascularly. Intravascular sickling and haemolysis lead to a cascade of events, including ischemia-reperfusion injury, the alteration of adhesion molecules, the activation of platelets and the coagulation cascade and nitric oxide (NO) depletion, ultimately causing endothelial damage, which results in the systemic vasculopathy and chronic inflammatory state that characterise SCD [2]. SCD is associated with a multitude of acute and chronic complications leading to significant morbidity, poor quality of life and reduced life expectancy. In addition, it results in the high utilisation of hospital facilities and overall healthcare resources [3].

Even though the correlation between SCD and multisystem complications is well known, a lot less is known about the dental complications for these patients, with two recent reviews showing conflicting results [4,5]. Here, we aim to explore the incidence of severe dental disease in the SCD population as well as identify if particular subgroups are at a higher risk.

## 2. Patients/Methods

This was a three-month audit exploring severe dental complications in patients with SCD. Dental extraction/loss was chosen as a composite measure of severe dental disease. Traumatic tooth loss or wisdom teeth extraction were excluded.

The patients with SCD in our institution were approached over a three-month period and asked to respond to a questionnaire regarding their dental health with emphasis on their history of dental extractions, the number of teeth lost and their age at first extraction (the full questionnaire can be found in Supplementary File S1). The patients were approached during routine outpatient appointments or before discharge from the hospital after an acute presentation and were asked to provide verbal consent for their data to be anonymously published. No specific selection criteria, such as age or genotype, were applied.

Clinical and laboratory data were subsequently retrieved from the patient’s electronic patient record (EPR) in order to identify the potential correlations between severe dental disease and their history of previous sickle-related complications as well as laboratory markers, including baseline Hb, markers of haemolysis such as reticulocyte count, bilirubin and lactic dehydrogenase (LDH). Once we calculated the median number of teeth lost and age at first extraction with compared groups less affected and those worse affected than average. Finally, the radiological findings for the subset of patients who had any procedures performed in our institution were also analysed.

Statistical analysis of the statistical difference between the binary variables described, i.e., the presence or absence of certain clinical characteristics, was achieved using Fisher’s Exact Test, with a *p*-value cut-off of <0.05.

Analysis of the statistical differences between the continuous and discrete variables, i.e., the laboratory findings, was by the use of Student’s *t*-test (for haemoglobin in the D.E. vs. No D.E. test) and by the Mann–Whitney test for all other variables, after analysis by the Shapiro–Wilcox test determined that the haemoglobin for the D.E. vs. No D.E. comparison was the only normally distributed variable; all others were not normally distributed. The outliers have been retained; however, removing them did not significantly alter the results. Statistical analysis was achieved using the R programming language (R Core Team (2022), R foundation for Statistical Computing, Vienna, Austria, https://www.R-project.org accessed on 15 July 2022) (for most testing), and Microsoft Excel (Microsoft Corporation (2018), One Microsoft, Way, Washington, WA, USA, https://office.microsoft.com/excel, accessed on 15 July 2022) (for Fisher’s Exact Test).

## 3. Results

### 3.1. Survey

One hundred eighty-two patients were approached in the three-month period of February–April 2022, of whom 177 provided consent to be interviewed and for their data to be collected and published. Seventy-four patients (42%) were male, and one hundred three patients (58%) were female. Overall, 121 patients (68%) had homozygous disease (Hb SS) or HbSβ^0^ while 56 (32%) had Hb SC or other genotypes. The mean age of the participating patients at the time of completing the questionnaire was 42 (range 17–87).

One hundred twenty-five patients (71%) had at least one dental extraction (DE) in their lifetime, with a median number of teeth extracted of three (1–all); it should be noted that the median figure does not include the two patients who had all of their teeth removed. The median age at first dental extraction was 26, with a mean of 25 (range 5–70).

Fifty-six of the 125 patients (45%) had all of their procedures performed at a dental practice in the community, and 43 patients (34%) had all procedures performed by oral and maxillofacial surgeons in hospital, 19 (44%) of whom in our own institution, while the remaining 26 patients (21%) had extractions both in hospital and the community. Overall, 32 patients (26%) had at least one dental extraction in our hospital.

Overall, 133 patients (75%) are registered with a dentist and 46 (25%) are not. However, only 81 patients (46%) answered positively when asked if they have regular dental check-ups.

### 3.2. Clinical Correlations

The male patients appeared to have a higher incidence (76%) of DE compared to females (67%) irrespective of genotype, but that did not reach statistical significance. There was no significant difference between homozygous patients and patients with Hb SC/other genotypes with a DE incidence of 70% and 74%, respectively. There were no significant differences in the incidence of DE in relation to the frequency of vaso-occlusive crises (VOC) or a previous history of acute chest syndrome (ACS), avascular necrosis, priapism, cerebral vascular disease, leg ulcers or sickle cell nephropathy and pulmonary arterial hypertension. Equally, within the subgroup of patients who had DE, there was no correlation between the above clinical parameters and the severity of dental disease, i.e., the number of teeth lost or the age at first extraction (Table 1). The incidence of DE for patients on treatment with hydroxyurea or chronic transfusions was 78% and 73%, respectively.

### 3.3. Haematological/Biochemical Parameters

**Figure 1:**
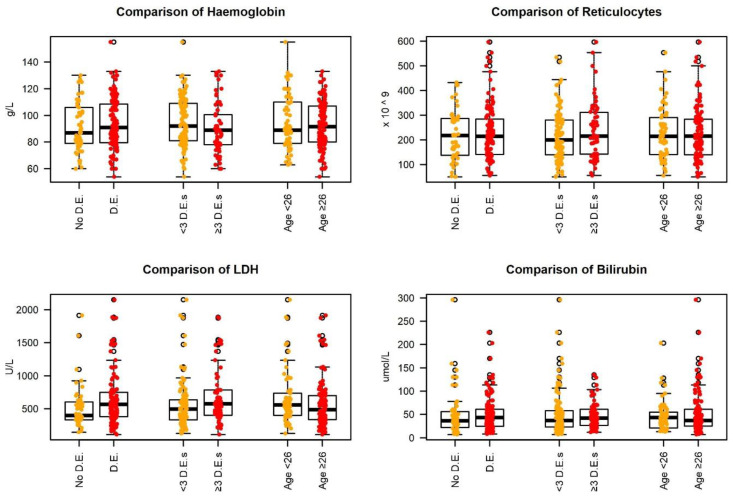
Correlation between dental extraction and haematological and haemolytic markers. D.E. is ‘Dental Extraction’, <3 and ≥3 refer to the number of extractions, and <26 and ≥26 refer to the age of the patient at their first extraction.

Baseline Hb did not differ significantly between patients who had or did not have DE, neither did it distinguish patients with dental extractions worse affected. The same lack of correlation was found for haemolytic markers, such as reticulocyte count and bilirubin). LDH did display some significant statistical differences between the groups of DE and No DE, and for the groups who had less than three dental extractions and those who had more than three (Figure 1).

### 3.4. Radiological Findings

The radiological data were available for 34 patients. The most common dental pathology reported in the X-rays was dental caries, with 21 (62%) affected patients. Of note, dental pathology was more common in the lower rather than the upper teeth, with 23 (68%) and 11 (32%) patients affected, respectively.

## 4. Discussion

This is a single-centre snapshot survey/audit looking into dental disease in SCD. As such, it has several limitations, including the size of the sample as well as the lack of detail around dental health problems that was necessary to make this survey easy for participation. Dental extraction was chosen as a composite index of severe dental disease without taking into consideration other more detailed means of assessing dental health.

Patients with SCD have been found to suffer from enamel defects such as hypoplasia or calcification, which in turn predispose to the development of caries [6]. Al-Alawi et al. found a significantly higher incidence of tooth decay in patients with SCD compared to controls even though the decayed, missing, and filled teeth (DMFT) index, the community periodontal index (CPI) or the plaque index [7] were not significantly higher. The mean age of the sample group in this study was lower than ours (24.5 versus 42 years). Another study comparing patients with β thalassaemia, SCD and controls found a significantly higher prevalence of caries and periodontal disease in patients with SCD compared to controls, including a higher DMFT index [8]. Similar results were found in a study involving children and adolescents with SCD when compared to controls [9].

SCD is characterised by recurrent episodes of severe pain, while it can also lead to severe, even life-threatening, acute or chronic complications. In this context, dental health may often be overlooked by patients and healthcare professionals looking after them alike.

In our study, we find almost 3/4 of patients with SCD require at least one dental extraction starting at a young age (median 26) and often requiring this intervention multiple times (median tooth loss: 3). We found no association between severe dental disease and the severity of the underlying SCD; homozygous and double heterozygous patients had similar incidence whilst severe dental disease did not differ according to the history of severe sickle-related complication including frequent painful VOCs and did not appear to be affected by baseline Hb or rate of haemolysis. Poor dental hygiene and lack of engagement with dental services have been highlighted as the main reason for the increased prevalence of dental disease observed in patients with SCD. Suffering from a chronic illness associated with episodes of severe pain may distract patients from realising the importance of dental care [8]. In fact, in the study by Al-Alawi et al., even though the number of decayed teeth was significantly higher in SCD patients compared to controls, the number of filled teeth were significantly lower, indicating a lack of appropriate and timely action in the face of dental problems [9]. Our findings support this hypothesis as we see no correlation with risk factors inherent to the underlying SCD.

All patients with SCD are under lifelong follow-up for monitoring of their condition. In the UK, among other routine consultations, patients are expected to have a specialist annual review, the results of which are entered into a national register (National haemoglobinopathy register). However, dental health/problems are not included in this “checklist”.

## 5. Conclusions

Dental disease and tooth loss impact the quality of life of patients with SCD, and the early identification of dental problems should be part of the management of these patients both in primary and specialist care.

Finally, the high prevalence of dental disease among SCD patients who are already burdened with a severe chronic physically and mentally distressing illness makes a strong argument for free dental care available to this patient group.

## Figures and Tables

**Table 1:** Association between dental extraction and sickle cell clinical characteristics.

	Total	DE n	DE %	*p*
SS	120	84	70	0.86
SC/OTHER	57	42	74	
10+ VOC	33	24	73	0.42
<10 VOC	144	99	69	
ACS	46	35	76	0.35
NO ACS	131	88	67	
AVN	41	29	71	1.0
NO AVN	136	95	70	
PRIAPISM	24	17	71	1.0
NO PRIAPISM	50	36	72	
CVA	17	11	65	0.59
NO CVA	160	113	71	
PAH	9	8	89	0.28
NO PAH	168	114	68	
LU	20	13	65	0.61
NO LU	157	111	71	
SCN	20	15	75	0.8
NO SCN	157	109	69	

DE = dental extraction, SS = Hb SS, SC = Hb SC, VOC = vaso-occlusive crisis, ACS = acute chest syndrome, CVA = cerebral vascular accident, PAH = pulmonary arterial hypertension, LU = leg ulcers, SCN = sickle cell nephropathy.

## Data Availability

The data presented in this study are available on request from the corresponding author. The data are not publicly available due to being part of departmental audit.

## Supplementary Materials

The following supporting information can be downloaded at: www.mdpi.com/article/10.3390/jcm11206174/s1. Supplementary File S1: questionnaire.
