# Supplementary material for: Rate of Dental Extractions in Patients with Sickle Cell Disease
Source: J Clin Med. 2022 Oct 19;11(20):6174. doi: 10.3390/jcm11206174 (PMC9605118; doi:10.3390/jcm11206174)
Supplement: Supplementary file 1 [file jcm-11-06174-s001.zip › jcm-1890586-supplementary.pdf]

Supplementary File S1

<u>Survey questionnaire</u>

1. Have you ever had a dental extraction?
Yes / No

2. How many teeth have you lost?
Number

3. At what age did you had your first dental extraction?
Number

4. Where did you have your dental extraction/s?
Community dental practice / Hospital–Homerton / Hospital–other /combination

5. Are you registered with a dentist?
Yes / No

6. Do you have regular dental check-ups?
Yes / No
